# The possible role furin and furin inhibitors in endometrial adenocarcinoma: A narrative review

**DOI:** 10.1002/cnr2.1920

**Published:** 2023-11-28

**Authors:** Hayder M. Al‐kuraishy, Thabat J. Al‐Maiahy, Ali I. Al‐Gareeb, Athanasios Alexiou, Marios Papadakis, Hebatallah M. Saad, Gaber El‐Saber Batiha

**Affiliations:** ^1^ Department of Clinical Pharmacology and Medicine College of Medicine, Mustansiriyah University Baghdad Iraq; ^2^ Department of Gynecology and Obstetrics College of Medicine, Mustansiriyah University Baghdad Iraq; ^3^ University Centre for Research & Development Chandigarh University, Chandigarh‐Ludhiana Highway Mohali Punjab India; ^4^ Department of Research & Development Funogen Athens Greece; ^5^ Department of Research & Development AFNP Med Wien Austria; ^6^ Department of Science and Engineering Novel Global Community Educational Foundation Hebersham New South Wales Australia; ^7^ Department of Surgery II University Hospital Witten‐Herdecke, University of Witten‐Herdecke Wuppertal Germany; ^8^ Department of Pathology Faculty of Veterinary Medicine, Matrouh University Matrouh Egypt; ^9^ Department of Pharmacology and Therapeutics Faculty of Veterinary Medicine, Damanhour University Damanhour AlBeheira Egypt

**Keywords:** endometrial adenocarcinoma, furin, inflammatory signaling, invasion, metastasis, proliferation

## Abstract

**Background:**

Endometrial adenocarcinoma (EAC) is a malignant tumor of the endometrium. EAC is the most common female malignancy following the menopause period. About 40% of patients with EAC are linked with obesity and interrelated with hypertension, diabetes mellitus, and high circulating estrogen levels. Proprotein convertase (PC) furin was involved in the progression of EAC.

**Recent findings:**

Furin is a protease enzyme belonging to the subtilisin PC family called PC subtilisin/kexin type 3 that converts precursor proteins to biologically active forms and products. Aberrant activation of furin promotes abnormal cell proliferation and the development of cancer. Furin promotes angiogenesis, malignant cell proliferation, and tissue invasion by malignant cells through its pro‐metastatic and oncogenic activities. Furin activity is correlated with the malignant proliferation of EAC. Higher expression of furin may increase the development of EAC through overexpression of pro‐renin receptors and disintegrin and metalloprotease 17 (ADAM17). As well, inflammatory signaling in EAC promotes the expression of furin with further propagation of malignant transformation.

**Conclusion:**

Furin is associated with the development and progression of EAC through the induction of proliferation, invasion, and metastasis of malignant cells of EAC. Furin induces ontogenesis in EAC through activation expression of ADAM17, pro‐renin receptor, CD109, and TGF‐β. As well, EAC‐mediated inflammation promotes the expression of furin with further propagation of neoplastic growth and invasion.

## 1 INTRODUCTION

Endometrial adenocarcinoma (EAC) is a malignant tumor of the endometrium causing abnormal vaginal bleeding, dyspareunia, pelvic pain, and dysuria.^1^ EAC is the most common female malignancy following the menopause period and presented with irregular vaginal bleeding and clear vaginal discharge.^1^ About 40% of patients with EAC are linked with obesity.^1^, ^2^ As well, EAC is interrelated with hypertension, diabetes mellitus, and high circulating estrogen level.^3^ EAC is the third greatest common cause of death in women following ovarian and cervical cancers.^1^, ^3^ EAC is the most common malignant tumor in developed countries and represents 80% of endometrial cancer.^4^ Remarkably, the rate of EAC has been shown to increase between 1980 and 2020 due to the growing rate of obesity and the increasing rate of elderly women.^4^

Regarding the molecular classification of EAC, recent studies illustrated four types including DNA polymerase epsilon (POLE) ‐mutated, copy number low, copy number high, and hypermutated type.^5^, ^6^ According to analogous classification system, five types are recorded including POLEmut, mismatch repair (MMR) deficient, p53 aberrant, and no specific molecular profile.^5^, ^6^ The National Comprehensive Cancer Network has integrated molecular analysis into its endometrial carcinoma algorithm as a result of the increasing amount of evidence that supports this classification system.^5^

Diagnosis of EAC is done by endometrial biopsy.^4^ Molecular biomarkers like DNA ploidy, hormone receptor, p53 level, stathmin, and level of adhesion molecules have been used in the diagnosis and prognosis of EAC.^4^ Due to the heterogeneity of EAC, many molecular biomarkers might be not enough for a more accurate diagnosis of this type of malignancy. These methods are not “used” but they were “examined” as a screening serum marker.^7^ Diagnosis should be confirmed by histopathologic examination of the endometrial biopsy, and it is the golden standard, and it can be applied as an office biopsy. Currently, there is no molecular diagnostic marker for any disease to replace histopathology. However many molecular markers are under examination for prognostic or therapeutic purposes mainly in EAC. This molecule may be high in endometrial cancer, however, to describe a diagnostic marker, and to report the presence or absence of a condition (here endometrial cancer) we need an accuracy test that describes the sensitivity, specificity, predictive values, and the likelihood ratios.^8^ In order to render clinical judgments and direct the provision of patient treatment, healthcare professionals must possess a comprehensive grasp of the probability of a patient's affliction, achieved through the amalgamation of their understanding of pretest probability and diagnostic evaluations. Diagnostic instruments are regularly employed in healthcare environments for the purpose of ascertaining appropriate courses of action; nevertheless, numerous of these instruments are susceptible to fallacy.^8^ The identification of EAC is conducted through the use of an endometrial biopsy.^4^ Due to the heterogeneity of EAC, many molecular biomarkers might be not enough for a more accurate diagnosis of this type of malignancy. To promote the progress of clinical outcomes in women with EAC, numerous strategies are suggested to reach the primitive diagnosis of EAC particularly in the early stage.^9^ Indeed, some biomarkers like micro‐RNA, miR223, miR222, and CA‐125 can accurately differentiate high and low‐risk women with EAC.^9^ It has been shown that proprotein convertase (PC) furin was involved in different tumor progressions including EAC.^10^ Different studies revealed that furin was the unique PC type linked with the development of EAC as confirmed by endometrial biopsies in postmenopausal women.^11^, ^12^ Thus, furin could be a noninvasive tool in the diagnosis of EAC. The rationale of the present study was regarded as due to the involvement of furin in various types of malignancies.^13^ Therefore, furin could be a noninvasive tool in the diagnosis of EAC. Therefore, this review aimed to find the association between furin activity and EAC.

## 2 FURIN PATHWAY OVERVIEW

Furin is a protease enzyme also named paired basic amino acid cleaving enzyme encoded by the furin gene, involved in the activation of several proteins.^14^ Furin belongs to subtilisin/kexin type 3 converts precursor proteins to biologically active forms and products.^14^ The substrates of furin are pro‐albumin, pro‐parathyroid hormone, pro‐beta‐secretase, transforming growth factor beta‐1 (TGFβ‐1), von Willebrand factor, and type 1 matrix metalloproteinase.^15^ As well, furin is implicated in the pathogenesis of juvenile hemochromatosis by activation of the hemojuvelin gene.^15^ Furin is highly expressed in the Golgi apparatus where it cleaves inactive proteins to their active forms.^15^ Furin is also concerned in the activation of virus envelop proteins as in human immune deficiency virus, dengue fever, influenza virus, Ebola virus, Marburg virus, and severe acute respiratory syndrome coronavirus type 2 (SARS‐CoV‐2).^16^ Furin is also engaged in the proteolytic activation of pseudomonas and anthrax toxins.^14^, ^17^

**FIGURE 1:**
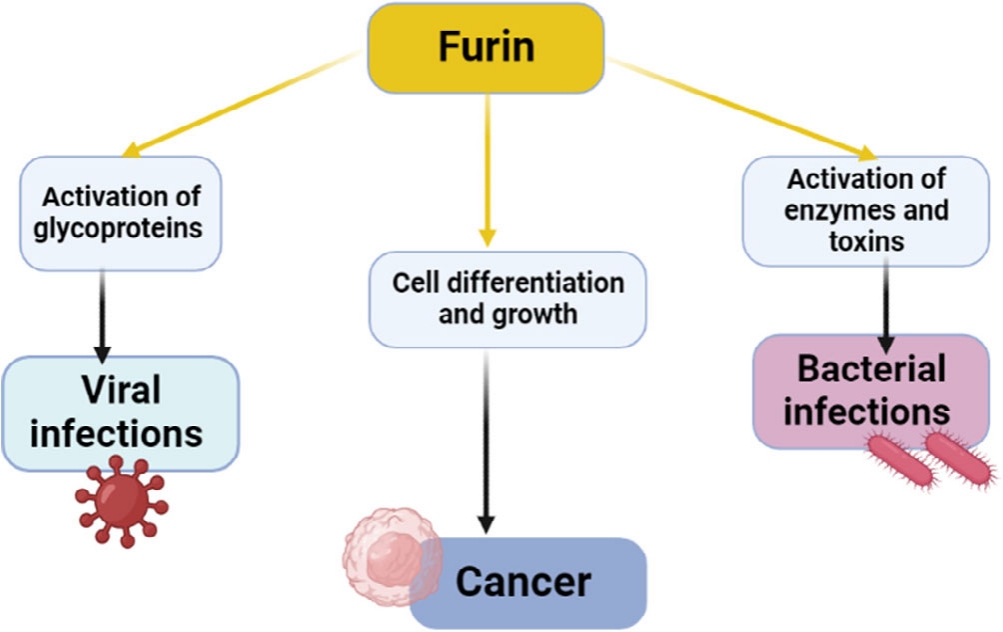
Role of proprotein convertase furin in the development of cancer and viral/bacterial infections.

Variations and defects in the expression and enzyme activity of furin promote the progression of a wide range of diseases including dementia, rheumatoid arthritis, and cancer.^18^, ^19^ Moreover, furin has a role in the activation differentiation and growth of human cells.^19^ Aberrant activation of furin promotes abnormal cell proliferation and the development of cancer. PC furin increases the pathogenesis of viral and bacterial infections (Figure 1).

## 3 FURIN AND CARDIOMETABOLIC DISORDERS

It has been shown that furin is associated with development of cardiometabolic disorders including obesity and type 2 diabetes mellitus (T2DM).^20^, ^21^ Furin enhances the levels of high‐density lipoprotein by deactivating lipoprotein lipases in the endothelium.^22^ The cleavage of membrane‐bound transcription factors, specifically referred to as sterol regulatory element binding proteins, by furin leads to the increase in the synthesis of sterols, lipids, and the LDL receptor.^22^ Furin is responsible for the reduction of protein levels of the LDL receptor by amplifying its intracellular metabolic pathway in acidic compartments within the subcellular environment.^22^ Furthermore, hepatic furin diminishes the functionality of endothelial lipase by means of stimulating the cleavage of angiopoietin‐like protein 3, which is an innate inhibitor of endothelial lipase.^23^ Therefore, the act of inhibiting furin in the liver leads to an increase in the activity of endothelial lipase, which subsequently causes a reduction in HDL‐C levels and hampers the process of reverse cholesterol transport.^23^ The hepatic furin pathway represents a newly discovered mechanism that controls the metabolism of high‐density lipoproteins (HDL) and the maintenance of cholesterol homeostasis.^23^ The results of this study imply that furin plays a role in lipid metabolism and the development of dyslipidemia.

A population‐based prospective study involved subjects at high risk for the development of T2DM and revealed that initial high furin serum levels increased the risk for the development of T2DM and premature mortality.^20^, ^24^ Mutation in furin induces propagation of insulin resistance (IR) and T2DM due to association with mutation in the insulin pro‐receptors.^25^ Indeed, high circulating furin serum level is correlated with dyslipidemia, high body mass index, and development of metabolic syndrome.^26^ Moreover, genetic variation of furin is connected with the development of systemic hypertension.^27^ A cohort study that included 94 hypertensive patients showed that single nucleotide polymorphism of furin was associated with the development and severity of hypertension.^27^

In contrast, a longitudinal study comprised Chinese adults with hypertension illustrated that low furin levels were associated with a high risk of hypertension^28^ suggesting a protective effect of furin. Similarly, a prospective study demonstrated that a deficiency of furin increases the risk for the development of abdominal obesity.^29^, ^30^, ^31^ However, Ren et al.^32^ disclosed that furin through induction of inflammatory reaction and lipid dysmetabolism may promote the propagation of atherosclerosis and hypertension. These findings suggest a latent controversy regarding the role of furin in the development of cardiometabolic disorders. Recently, cardiometabolic disorders have been associated with the development and progression of different cancer types including EAC.^33^ It has been observed that obesity is regarded as an established risk factor for EAC, and weight reduction in obese women by bariatric surgery decreases EAC risk by 81%.^34^ In addition, the use of combined oral contraceptives protects against EAC for about 30 years.^34^ The mechanisms of obesity‐induced EAC are not fully elucidated, though adipose‐derived estrogen, which is unopposed by progesterone in postmenopausal obese women could be the potential mechanism.^35^ Besides, T2DM and IR promote the development of EAC due to dysregulation of insulin signaling and adipocytokines.^36^ However, a systematic review and meta‐analysis illustrated that insulin‐sensitizing metformin improves survival in patients with EAC, but does not prevent endometrial proliferation after 2–16 weeks of treatment.^37^ Furthermore, hormonal changes are increased at menopause characterized by reducing estrogen levels, which cause loss of subcutaneous adipose tissue and augment visceral fat.^38^, ^39^ However, adipose tissues remain the major source of estrogen by converting androgen derived from ovary and adrenal gland to estrogen via aromatase enzyme, which is highly expressed in adipose tissue.^38^, ^39^ Therefore, hormonal changes in menopause and the development of visceral obesity together with cardiometabolic may increase EAC through furin‐dependent mechanisms.

## 4 FURIN AND TUMOR PROGRESSION

Furin is considered a master switch and regulator of tumor cell proliferation and progression.^40^ The expression of aberrant furin triggers the development and progression of different types of malignancies including lung carcinoma, rhabdomyosarcoma, colonic cancer, and skin cancers.^10^, ^40^ It has been reported that furin was regarded as a potential biomarker of cancer staging since it correlated with the aggressiveness of cancer type.^40^ Furin promotes angiogenesis, malignant cell proliferation, and tissue invasion by malignant cells through its pro‐metastatic and oncogenic activities.^40^ In addition, furin through activation of numerous growth factors increases cell proliferation and tumor growth.^10^, ^40^ For example, furin promotes the activation of vascular endothelial growth factor and lymphangiogenic factor which increase the vascularization of solid tumors.^40^

Of note, furin also increases the proliferation and vascularization of tumors in hypoxic areas. Tissue hypoxia induces the expression of hypoxia‐inducible factor 1 alpha, which triggers the induction of furin in hypoxic tissues.^41^ Also, hypoxia enhances intracellular localization of furin with subsequent direct interaction and activation of oncogenic growth factors.^41^ Thus, hypoxia may increase the proliferation and growth of malignant cells through the furin‐dependent pathway.

Furthermore, furin can increase metastasis of malignant cells through induction the expression of integrin and matrix metalloproteinases (MMPs), which assist metastasis by degrading the extracellular matrix.^42^ Notably, there is a close interaction between furin and interferon‐gamma (INF‐γ), which increase the expression of PC furin. Castro et al.^43^ pointed out that INF‐γ may have a pro‐tumorigenic role through the downregulation expression of major histocompatibility complexes, also it acts as a checkpoint inhibitor promoting immunosuppressive microenvironment in the tumor area. Furthermore, furin increases the expression of INF‐γ during tumor growth and invasion.^44^

Interestingly, furin attenuates cell antitumor activity by promoting the development of immunotolerant and immunosuppressive T cells namely regulatory T cells with inhibition of cytotoxic T cells.^45^ Besides, activated immune cells as activated T cells demonstrated a higher expression of furin.^45^ As well, infiltration of tumors by the immune cells is correlated with the expression of furin. In this state, deficiency of furin may be associated with the reduction in the infiltration of the tumor by immunosuppressive regulatory T cells leading to a better immunological response.^45^, ^46^ Herein, the inactivation of furin in the activated T cells may inhibit tumor growth and invasion.^46^ Therefore, targeting furin could be a potential therapeutic value in the management of cancer.^40^ However, PC furin inhibitors may increase the aggressiveness of some human cancers.^40^

In vitro study, included ovarian cancer cell lines demonstrated that furin was highly expressed^47^ suggesting that furin could be a biomarker of ovarian carcinoma.^47^ Chen and colleagues observed that furin induces proliferation and metastasis of ovarian cancer through stimulation of signal transducer activator transduction 3.^48^, ^49^ Moreover, in vitro and in vivo studies revealed that overexpression of furin was correlated with the risk of cancer development.^50^, ^51^

Moreover, furin activity is increased in both atypical endometrial hyperplasia and endometriosis.^10^, ^30^ Total furin activity in cervical swabs and uterine lavage correlated with the severity and progression of atypical endometrial hyperplasia and endometriosis.^10^, ^30^ An experimental study illustrated that inhibition growth of endometriosis by baicalein reduced furin level.^52^ The results imply the possible function of PC furin in the propagation of atypical endometrial hyperplasia and endometriosis.

**FIGURE 2:**
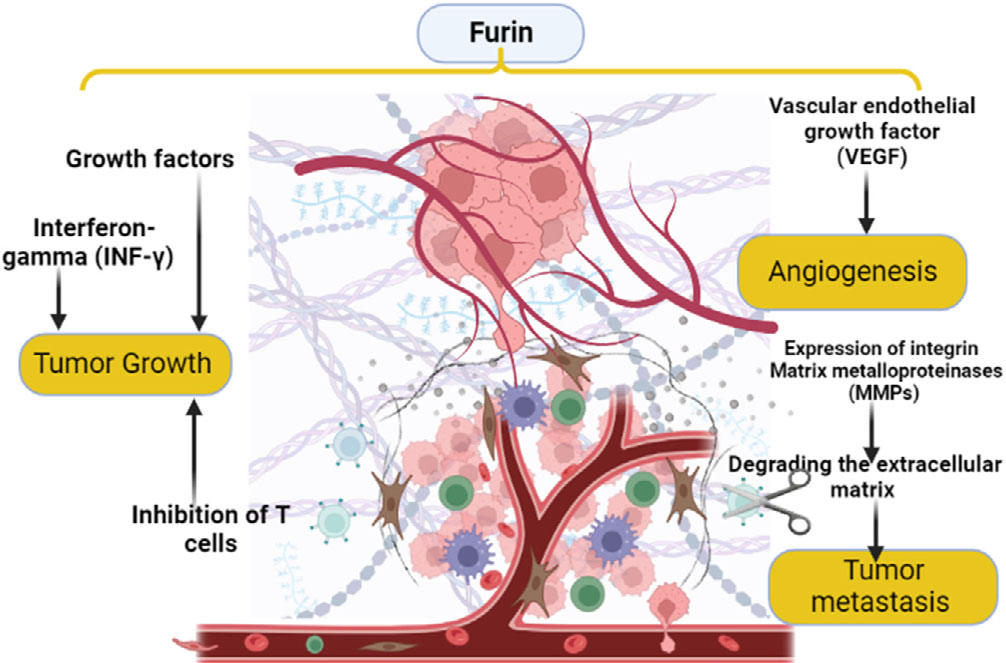
The role of furin in tumor progression: furin activates expression of growth factors which stimulate cell proliferation; furin increases metastasis by increasing the expression of integrin and matrix metalloproteinases (MMPs), also furin reduces cellular antitumor activity by inhibition of T cells. These changes trigger tumor progression.

These verdicts proposed that aberrant activation of furin is correlated with malignant proliferation and metastasis of different mammalian cancers (Figure 2).

## 5 FURIN AND EAC

Low‐grade EAC is characterized by well‐differentiated cells not invading the myometrium.^1^ However, high‐grade EAC is less differentiated and associated with atrophied endometrium.^3^ Some EAC have many foci of mucinous changes, which increase the severity and invasiveness of EAC.^1^, ^3^ Of note, furin activity is correlated with the malignant proliferation of EAC.^10^ A direct link between furin activity and the development of EAC was less assessed in the clinical studies. However, a recent study confirmed the association between a higher expression of TGF‐β and the progression of EAC.^53^ Particularly, furin is regarded as a TGF‐β converting enzyme that increase the oncogenic activity of TGF‐β.^54^ In addition, plasminogen activator inhibitor 1 (PAI‐1) inhibits intracellular activation of furin thereby reducing derangement in metabolic syndrome.^55^, ^56^ Therefore, PAI‐1 attenuates intracellular communication of adiposity‐mediated development of EAC through inhibition of PC furin.^57^ Besides, PAI‐1 inhibits the expression of TGF‐β.^57^ Thus, a higher expression of PAI‐1 in obesity could be a protective mechanism against the development of EAC through suppression of furin/TGF‐β axis.^58^

Furthermore, furin induces the processing of CD109 into small proteins which form an intricate complex with type 1 TGF‐β receptor for the direction of TGF‐β signaling in cancer cells.^59^ It has been reported that expression of the CD109 gene was increased in patients with EAC, though this expression was higher in cervical squamous cell carcinoma compared to EAC.^60^ In general, the expression of the CD109 gene was elevated mainly in squamous cell carcinoma than in adenocarcinoma.^61^ A meta‐analysis study illustrated that the expression of the CD109 gene could be a beneficial biomarker of cancer in general.^62^ These observations suggest that the CD109 gene is not a specific biomarker in women with EAC.

Moreover, emerging evidence proposes a potential role of the pro‐renin receptor in the ontogenesis and pathogenesis of EAC. In vitro study used endometrial cancer cell lines demonstrated that the pro‐renin receptor was crucial for the development of EAC through maintaining endometrial cell viability.^63^ As well, the pro‐renin receptor is highly expressed in human EAC compared to normal endometrium.^63^ Pro‐renin receptor improves cell migration, proliferation, and angiogenesis, which are essential for tumorigenesis of EAC.^64^ Pro‐renin receptor promotes activation of the renin‐angiotensin system (RAS), increasing the production of angiotensin II.^65^, ^66^ Similarly, the pro‐renin receptor enhances cellular viability and proliferation through induction expression of growth factors like phosphatidylinositol 3 kinase p85α subunit (PI3K‐p85α).^65^, ^67^, ^68^, ^69^ Pringle et al.^70^ study demonstrated that women with EAC had mutant RAS compared to healthy women. Obesity and high estrogen level are implicated in the generation of mutant RAS with the development of EAC.^70^ Therefore, RAS inhibitors could be protective agents against the development of cancer in both males and females by inhibiting RAS/pro‐renin receptor axis‐mediated intracellular activation.^71^, ^72^ In this state, furin increases the activity and level of the soluble pro‐renin receptor by intracellular cleavage.^73^

**FIGURE 3:**
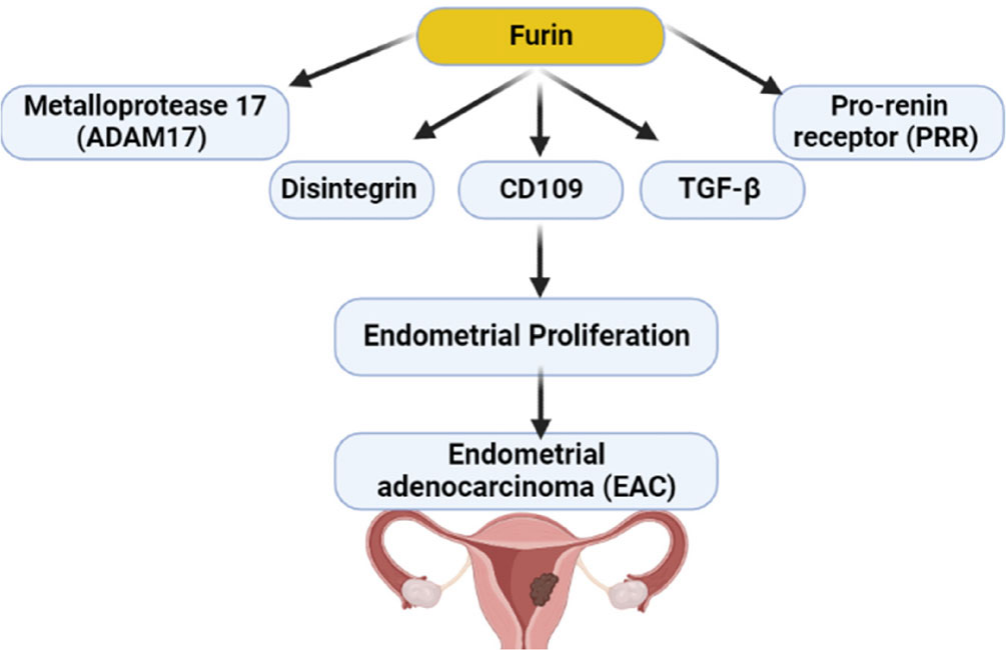
Role of furin in the development of endometrial adenocarcinoma (EAC): furin activates expression of transforming growth factor‐beta (TGF‐β), CD109, pro‐renin receptor (PRR) and disintegrin and metalloprotease 17 (ADAM17) with subsequent endometrial proliferation and EAC.

Indeed, disintegrin and metalloprotease 17 (ADAM17) also increase proteolytic activation of pro‐renin receptor.^73^ ADAM17‐mediated proteolysis of membrane proteins promotes intracellular signal transduction in cell proliferation, migration, and differentiation.^74^ ADAM17 increases the release of tumor necrosis factor‐alpha (TNF‐α) with induction of inflammation.^24^, ^75^, ^76^ ADAM17 is activated by PC furin with subsequent induction release of TNF‐α.^75^ Higher ADAM17 expression is linked with the development of EAC.^77^ Xu and coworkers found that a high level of ADAM17 was associated with poor clinical outcomes in women with uterine carcinoma.^78^ Therefore, higher expression of furin may increase the development of EAC through overexpression of pro‐renin receptors and ADAM17 (Figure 3).

Expression of furin was increased in postmenopausal endometrial biopsies as compared to controls.^11^ Uterine lavage is a noninvasive source material for evaluating the endometrium. Furin activity was altered in the uterine lavage of EAC patients as compared to controls.^11^ Furin activity was detected in all uterine lavage samples and significantly elevated in all grades of EAC.^11^ Notably, the expression of furin is variable according to the stages of EAC, being higher in the early stages and reduced with increasing grades of EAC.^11^ Endometrial biopsies from 30 women with EAC and 7 healthy controls showed that furin activity was higher in patients compared to controls, but this expression was reduced as the disease advanced.^11^ However, total PC activity of uterine lavage was increased in all grades and stages of EAC in women with EAC compared with healthy controls.^11^ These findings suggest that furin is more accurate than total PC in the diagnosis, staging, and prognosis of EAC, as a furin decrease indicates a poor prognosis.

Thus, monitoring the total PC activity in uterine lavage may provide a rapid and noninvasive method for the diagnosis of EAC in postmenopausal women.^11^ Heng et al.^30^ observed that furin activity was significantly increased in the uterine lavage of postmenopausal women with EAC. Uterine lavage can be acquired in a noninvasive manner in contrast to the procurement of uterine tissues; nonetheless, the broad clinical application of uterine lavage is constrained by blood contamination and various other factors. The correlation between furin activity in swabs and lavage was found to be remarkably significant in postmenopausal women. Moreover, there was a notable increase in furin activity within endocervical swabs in patients with EAC as compared to the control group.^30^ Hence, the furin activity observed in endocervical swabs could potentially serve as a straightforward, nonintrusive, and innovative approach for identifying EAC in women who have reached menopause.

## 6 FURIN AND INFLAMMATION IN EAC

It has been shown that furin has pro‐inflammatory and anti‐inflammatory effects in different inflammatory and metabolic disorders.^79^ Furin level is correlated with the level of pro‐inflammatory cytokines.^79^, ^80^ A comparative study illustrated that children with obesity had a higher rate of furin and pro‐inflammatory cytokine levels as compared to normal‐weight children.^79^ Inhibition of furin was shown to decrease vascular remodeling and systemic inflammation.^81^, ^82^ Therefore, a high furin level reflects underlying systemic inflammation and could be a potential link between obesity and inflammation.

In contrast, a previous study demonstrated that myeloid cells expressing furin had anti‐inflammatory effects through modulation of T cell immune response and tolerance.^83^, ^84^ Experimental studies revealed that deletion of furin promotes aberrant activation and polarization of T cells with the development of autoimmunity due to the lack of immune tolerance.^85^, ^86^ Lin et al.^87^ illustrated that furin had a protective role against inflammatory changes in patients with rheumatoid arthritis by regulating T cell immune tolerance. These verdicts suggest a pro‐inflammatory and anti‐inflammatory role of furin in different inflammatory conditions.

On the other hand, it has been hypothesized that chronic inflammation may promote the development of EAC through induction of rapid cell division; DNA injury; mutation and ineffective DNA repair.^2^, ^88^ High estrogen improves endometrial inflammation through the induction expression of prostaglandin E2 (PGE2) and cyclooxygenase‐2 (COX‐2), which induce tumorigenesis by increasing the generation of pro‐inflammatory cytokines including interleukin (IL)‐6 and IL‐8.^89^, ^90^ Besides, COX‐2 increases the generation of free radicals and reactive oxygen species (ROS) through the induction expression of nitric oxide synthase.^89^, ^91^ Thus, ROS and inflammatory reactions in the endometrium may induce neoplastic transformation and the development of EAC.^88^

Indeed, the inflammatory signaling pathway nuclear factor kappa B (NF‐κB) is involved in cell proliferation, apoptosis, and angiogenesis. NF‐κB provokes malignancy signaling in both tumor‐associated inflammatory and cancer cells.^92^, ^93^ Inhibition of the NF‐κB signaling pathway and associated inflammatory changes may decrease the risk of malignant transformation and progression of EAC.^94^ Of note, furin increases the expression of COX‐2, PGE2, and NF‐κB in the initiation of labor.^95^ Also, PGE2 and COX‐2 increase the expression of furin, and inhibition of COX‐2 and PGE2 can decrease the expression of PC furin in cancer cells.^95^ Interestingly, activated NF‐κB promotes the expression of furin in EAC.^96^ Therefore, nonsteroidal anti‐inflammatory drugs like aspirin could be effective in reducing the risk of EAC through inhibition of the NF‐κB/COX‐2 axis.^97^, ^98^ A meta‐analysis included seven case–control studies and eleventh cohort studies of 14 766 endometrial cases showed that aspirin was regarded as a protective factor against the development of EAC.^99^

**FIGURE 4:**
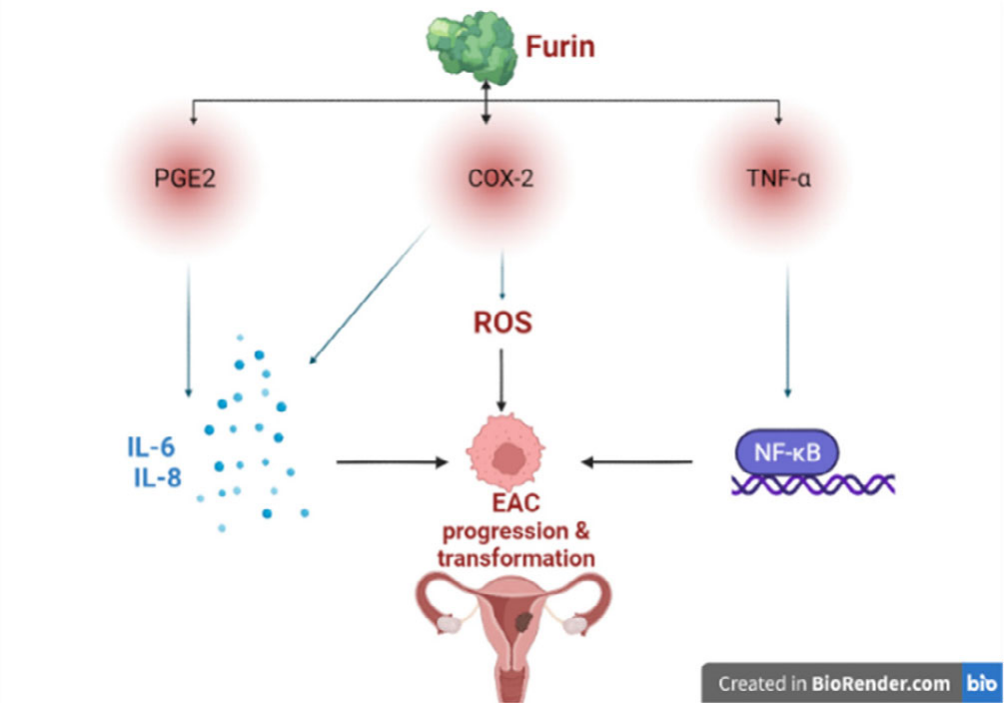
Inflammatory signaling in endometrial adenocarcinoma (EAC): EAC promotes the expression of proprotein convertase furin with further propagation of malignant transformation. COX‐2, cyclooxygenase‐2; NF‐κB, nuclear factor kappa B; PGE2, prostaglandin E2; ROS, reactive oxygen species; TNF‐α, tumor necrosis factor‐alpha.

These findings suggest that inflammatory signaling in EAC promotes the expression of furin with further propagation of malignant transformation (Figure 4).

## 7 FURIN INHIBITORS

Expression and activity of furin are essential for tumor progression and metastasis via activation of MMPs, which are correlated with tumor aggressiveness.^100^ Furin inhibitors could be of therapeutic value in inflammation, infection, and cancer.^101^ It has been reported that polyarginines are potent furin inhibitors.^101^ Small molecule compounds that inhibit furin and/or furin are of great value in cancer management.^100^ In vitro and vivo findings showed that furin inhibitor alpha‐1PDX reduced tumorigenicity in a considerable way.^100^ Despite evidence of efficacy in preclinical studies, no furin inhibitor is used clinically. Therefore, repurposing clinically FDA‐approved furin inhibitors is of great value.

### 7.1 Metformin

Insulin‐sensitizing drug metformin had been confirmed by preclinical in vitro study to inhibit furin and other genes associated with tumor progression and invasion.^102^ Of interest, in vitro invasion in EAC was considerably inhibited by sera from women with polycystic ovarian syndrome following 6 months of metformin treatment.^103^ This effect is mediated by the inhibition of MMP‐2/9 and furin.^103^ Other studies also confirmed the effectiveness of metformin against EAC.^104^, ^105^, ^106^, ^107^ In addition, metformin reverses obesity‐induced aggressiveness of EAC by inhibiting growth factors.^108^ It has been shown that metformin the proliferation of EAC as documented in an in vitro study in a dose‐dependent manner in progesterone resistance Ishikawa cells by inducing autophagy^108^ signifying that metformin is effective in progesterone resistance EAC. A randomized clinical trial started in 2019, showed that medroxyprogesterone acetate in combination with metformin is an effective therapeutic strategy against EAC.^105^ A systematic review and meta‐analysis showed that metformin in combination with progestin is more effective than progestin alone in the management of atypical endometrial hyperplasia and early EAC.^109^ Metformin can mitigate abnormal glucose metabolism, IR, and hyperinsulinemia, which are involved in the pathogenesis of EAC.^105^ In addition, medroxyprogesterone antagonizes the carcinogenic effect of estrogen,^105^ therefore this combination seems to be more appropriate in postmenopausal women with obesity and EAC. A clinical trial showed that long‐term metformin treatment can induce endometrial atrophy in 96% of women with endometrial hyperplasia.^110^ The anticancer mechanism is mediated by inhibiting inflammatory signaling pathways, such as mammalian target of rapamycin (mTOR) and mitogen‐activated protein kinase (MAPK), which are involved in the pathogenesis of EAC.^106^ A case–control study revealed that metformin inhibits the AKT/PI3K/mTOR signaling pathway in women with EAC compared to healthy controls.^107^ These findings indicated that metformin through inhibition of furin, inflammatory signaling pathways, and growth factors may be effective in treating EAC.

### 7.2 Statins

Statins are 3‐hydroxyl‐3‐methyl‐glutaryl coenzyme A (HMG‐CoA) reductase inhibit hepatic denovo cholesterol biosynthesis.^111^, ^112^ Statins are widely used in the management of hypercholesterolemia and dyslipidemia, therefore they are used in the prevention of primary and secondary cardiovascular complications. In addition, statins have anti‐inflammatory, antioxidant, anti‐thrombotic, and antiapoptotic effects.^113^ Moreover, clinical studies established that statins have the ability to inhibit furin activity.^114^, ^115^ Furin plasma levels are increased in patients with acute cardiac events and following cardiac intervention in statins‐free patients but reduced in statins‐treated patients.^106^, ^114^ Higher plasma levels of furin and PCSK9 in patients with acute cardiac events are associated with resistance to statins therapy.^115^ In addition, statins attenuate SARS‐CoV‐2 infection by inhibiting furin.^116^ Interestingly, high cholesterol increases the activity of furin for priming and activation of SARS‐CoV‐2 spike protein to bind angiotensin‐converting enzyme 2 (ACE2).^117^ Therefore, statins can directly inhibit furin or indirectly reduce furin activity by reducing cholesterol.

On the other hand, statins can improve the survival of women patients with EAC by inhibiting endometrial cell proliferation and migration through modulation of the expression of furin.^118^, ^119^ However, long‐term use of statins led to borderline or ineffectiveness against the development and progression of EAC.^120^ Statins have chemoprotective effects against EAC in high‐risk group women.^118^ Findings from the Australian record linkage study demonstrated a potential benefit for statins use in women with EAC.^119^ It has been reported women with EAC on statins therapy had a lower mortality rate compared to women not on statins therapy.^119^ A retrospective study involved 985 women with EAC showed that statins improved survival and reduce complications.^121^ In many epidemiological studies statins reduced EAC and ovarian risk,^122^ however, statins were reported to be effective for ovarian but not for EAC.^123^ However, a meta‐analysis and systematic review revealed that long‐term use of statins >5 years did not EAC risk.^124^ These findings suggest that statins via inhibition of furin could be effective as adjuvant treatments in the management of EAC.

### 7.3 Baicalein

Baicalein is a flavonoid isolated from *Scutellaria lateriflora* and *Scutellaria balacalensis*, has anti‐inflammatory, antioxidant, and anticancer effects.^125^ Baicalein is regarded as an allosteric modulator of benzodiazepine receptors, and inhibits lipooxygenase.^126^ It has been observed that baicalein has anticancer properties by numerous molecular mechanisms including inhibition of PI3K/AKT/mTOR, and MAPK.^127^ Furthermore, baicalein inhibits adenosine diphosphate ribosylation factor 6, which promotes the proliferation and invasion of endometrial cancer cells.^128^ Similarly, baicalein modulates the expression of progesterone receptors, which is intricate in the development of EAC.^129^ Furthermore, baicalein attenuates the carcinogenesis of EAC by inhibiting the expression of inflammatory signaling pathways.^130^ As baicalein is not clinically approved, therefore there are limitations regarding the clinical effects of baicalein in women with EAC.

The underlying mechanism of baicalein in suppressing proliferation and invasion of EAC is not fully elucidated. However, inhibition of endometrial furin by baicalein could a possible mechanism. Preclinical studies illustrated that baicalein can inhibit endometrial proliferation and ectopic endometriosis by inducing apoptosis and expression of MMP and furin.^52^, ^131^ Therefore, furin inhibitor baicalein according to the preclinical findings seems to be effective against EAC.

Taken together, furin inhibitors, such as metformin and statins could be a novel adjuvant therapeutic strategy in the management of EAC.

The present review had several limitations including a paucity of clinical studies and the sequential level of furin in the different stages of EAC was not reviewed. However, this review highlighted the potential role of furin and associated inflammatory changes in the propagation of EAC. These findings indicated that furin inhibitors produce borderline effects in EAC. Therefore, this review exciting researchers in this field to perform future clinical trials, pilot, and prospective studies to confirm the exact role of furin in the pathogenesis of EAC regarding the efficacy and safety of furin inhibtors.

## 8 CONCLUSIONS

EAC is the most common malignant tumor of the female genital tract and is linked with menopause, obesity, and other cardiometabolic disorders. Furin is associated with the development and progression of EAC through induction of proliferation, invasion, and metastasis of malignant cells of EAC. Furin induces EAC through activation expression of ADAM17, pro‐renin receptor, CD109, and TGF‐β. As well, EAC‐mediated inflammation promotes expression of furin with further propagation of neoplastic growth and invasion. Taken together, furin has direct and indirect role in the advancement of EAC. Therefore, experimental and clinical studies are reasonable in this state to verify the potential role of furin in the development of EAC.

## AUTHOR CONTRIBUTIONS

**Hayder M. Al‐kuraishy, Thabat J. Al‐Maiahy, Ali I. Al‐Gareeb, Athanasios Alexiou**: Conceptualization (lead); visualization; writing—original draft (lead). **Marios Papadakis**: writing—original draft (lead); funding acquisition (lead). **Hebatallah M. Saad, Gaber El‐Saber Batiha**: Conceptualization; supervision; resources (lead); writing—review and editing.

## CONFLICT OF INTEREST STATEMENT

The authors have stated explicitly that there are no conflicts of interest in connection with this article.

## ETHICS STATEMENT

Not applicable.

## CONSENT FOR PUBLICATION

Not applicable.

## Data Availability

Data sharing is not applicable to this article as no new data were created or analyzed in this study.

## References

[1] Horn L‐C , Höhn AK , Krücken I , Stiller M , Obeck U , Brambs CE . Mesonephric‐like adenocarcinomas of the uterine corpus: report of a case series and review of the literature indicating poor prognosis for this subtype of endometrial adenocarcinoma. J Cancer Res Clin Oncol. 2020;146(4):971‐983. 31927619 10.1007/s00432-019-03123-7 PMC11804491

[2] Batiha GE‐S , Gari A , Elshony N , et al. Hypertension and its management in COVID‐19 patients: the assorted view. Int J Cardiol Cardiovasc Risk Prev. 2021;11:200121. 34806090 10.1016/j.ijcrp.2021.200121 PMC8590508

[3] Kölbl AC , Schlenk K , Behrendt N , Andergassen U . The importance of hCG in human endometrial adenocarcinoma and brleast cancer. Int J Biol Markers. 2018;33(1):33‐39. 28967068 10.5301/ijbm.5000290

[4] Kit OI , Frantsiyants EM , Bandovkina VA , et al. Modifying effect of obesity on the content of sex hormones and their receptors in endometrial adenocarcinoma and its surrounding tissue. Cardiometry. 2022;21:34‐40.

[5] Alexa M , Hasenburg A , Battista MJ . The TCGA molecular classification of endometrial cancer and its possible impact on adjuvant treatment decisions. Cancers. 2021;13(6):1478. 33806979 10.3390/cancers13061478 PMC8005218

[6] Goulder A , Gaillard SL . Molecular classification of endometrial cancer: entering an era of precision medicine. J Gynecol Oncol. 2022;33(3):e47. 35443293 10.3802/jgo.2022.33.e47 PMC9024190

[7] Shreffler J , Huecker MR . Diagnostic Testing Accuracy: Sensitivity, Specificity, Predictive Values and Likelihood Ratios. StatPearls Publishing; 2020. 32491423

[8] Shreffler J , Huecker MR . Diagnostic Testing Accuracy: Sensitivity, Specificity, Predictive Values and Likelihood Ratios. StatPearls Publishing; 2022. 32491423

[9] Rižner TL . Discovery of biomarkers for endometrial cancer: current status and prospects. Expert Rev Mol Diagn. 2016;16(12):1315‐1336. 27817223 10.1080/14737159.2016.1258302

[10] Jaaks P , Bernasconi M . The proprotein convertase furin in tumour progression. Int J Cancer. 2017;141(4):654‐663. 28369813 10.1002/ijc.30714

[11] Singh H , Heng S , Nicholls PK , et al. Proprotein convertases in post‐menopausal endometrial cancer: distinctive regulation and non‐invasive diagnosis. Biochem Biophys Res Commun. 2012;419(4):809‐814. 22390935 10.1016/j.bbrc.2012.02.111

[12] Heng S , Stephens AN , Jobling TW , Nie G . Total PC activity is increased in uterine lavage of post‐menopausal endometrial but not ovarian cancer patients. J Cancer. 2016;7(13):1812‐1814. 27698920 10.7150/jca.16331 PMC5039364

[13] Basak A , Chen A , Scamuffa N , Mohottalage D , Basak S , Khatib A‐M . Blockade of furin activity and furin‐induced tumor cells malignant phenotypes by the chemically synthesized human furin prodomain. Curr Med Chem. 2010;17(21):2214‐2221. 20459383 10.2174/092986710791331040

[14] Braun E , Sauter D . Furin‐mediated protein processing in infectious diseases and cancer. Clin Transl Immunol. 2019;8(8):e1073. 10.1002/cti2.1073 PMC6682551 31406574

[15] Silvestri L , Pagani A , Camaschella C . Furin‐mediated release of soluble hemojuvelin: a new link between hypoxia and iron homeostasis. Blood. 2008;111(2):924‐931. 17938254 10.1182/blood-2007-07-100677

[16] Alsubaie N , Al‐kuraishy HM , Al‐Gareeb AI , et al. Statins use in Alzheimer disease: bane or boon from frantic search and narrative review. Brain Sci. 2022;12(10):1290. 36291224 10.3390/brainsci12101290 PMC9599431

[17] Al‐kuraishy HM , Al‐Gareeb AI , Alkazmi L , Habotta OA , Batiha GE‐S . High‐mobility group box 1 (HMGB1) in COVID‐19: extrapolation of dangerous liaisons. Inflammopharmacology. 2022;30:1‐10. 35471628 10.1007/s10787-022-00988-y PMC9040700

[18] Thomas G . Furin at the cutting edge: from protein traffic to embryogenesis and disease. Nat Rev Mol Cell Biol. 2002;3(10):753‐766. 12360192 10.1038/nrm934 PMC1964754

[19] Saad HM , Tourky GF , Al‐kuraishy HM , et al. The potential role of MUC16 (CA125) biomarker in lung cancer: a magic biomarker but with adversity. Diagnostics. 2022;12(12):2985. 36552994 10.3390/diagnostics12122985 PMC9777200

[20] Fernandez C , Rysä J , Almgren P , et al. Plasma levels of the proprotein convertase furin and incidence of diabetes and mortality. J Intern Med. 2018;284(4):377‐387. 29888466 10.1111/joim.12783 PMC6175079

[21] Hussien NR , Al‐Naimi MS , Rasheed HA , Al‐kuraishy HM , Al‐Gareeb AI . Sulfonylurea and neuroprotection: the bright side of the moon. J Adv Pharm Technol Res. 2018;9(4):120‐123. 30637228 10.4103/japtr.JAPTR_317_18 PMC6302683

[22] Seidah NG , Khatib AM , Prat A . The proprotein convertases and their implication in sterol and/or lipid metabolism. Biol Chem. 2006;387(7):871‐877. 16913836 10.1515/BC.2006.110

[23] Jin W , Wang X , Millar JS , et al. Hepatic proprotein convertases modulate HDL metabolism. Cell Metab. 2007;6(2):129‐136. 17681148 10.1016/j.cmet.2007.07.009 PMC2565575

[24] Rasheed HA , Al‐kuraishy HM , Al‐Gareeb AI , Hussien NR , Al‐Nami MS . Effects of diabetic pharmacotherapy on prolactin hormone in patients with type 2 diabetes mellitus: bane or boon. J Adv Pharm Technol Res. 2019;10(4):163‐168. 31742116 10.4103/japtr.JAPTR_65_19 PMC6844004

[25] Coppola I , Brouwers B , Meulemans S , Ramos‐Molina B , Creemers JW . Differential effects of furin deficiency on insulin receptor processing and glucose control in liver and pancreatic β cells of mice. Int J Mol Sci. 2021;22(12):6344. 34198511 10.3390/ijms22126344 PMC8231939

[26] Harlid S , Myte R , Van Guelpen B . The metabolic syndrome, inflammation, and colorectal cancer risk: an evaluation of large panels of plasma protein markers using repeated, prediagnostic samples. Mediators Inflamm. 2017;2017:1‐9. 10.1155/2017/4803156 PMC5381203 28522899

[27] Li N , Luo W , Juhong Z , et al. Associations between genetic variations in the FURIN gene and hypertension. BMC Med Genet. 2010;11(1):1‐7. 20707915 10.1186/1471-2350-11-124 PMC2936893

[28] He Y , Ren L , Zhang Q , et al. Serum furin as a biomarker of high blood pressure: findings from a longitudinal study in Chinese adults. Hypertens Res. 2019;42(11):1808‐1815. 31253944 10.1038/s41440-019-0295-6

[29] He Y , Ren L , Zhang Q , et al. Deficient serum furin predicts risk of abdominal obesity: findings from a prospective cohort of Chinese adults. Postgrad Med J. 2021;97(1146):234‐238. 32114491 10.1136/postgradmedj-2019-137422

[30] Heng S , Stephens AN , Jobling TW , Nie G . Measuring PC activity in endocervical swab may provide a simple and non‐invasive method to detect endometrial cancer in post‐menopausal women. Oncotarget. 2016;7(29):46573‐46578. 27374098 10.18632/oncotarget.10287 PMC5216818

[31] Al‐Nami MS , Al‐kuraishy HM , Al‐Gareeb AI , Al‐Mamoori F . Metabolic profile and prolactin serum levels in men with type 2 diabetes mellitus: old‐new rubric. Int J Crit Illn Inj Sci. 2019;9(3):120‐126. 31620350 10.4103/IJCIIS.IJCIIS_40_19 PMC6792395

[32] Ren K , Jiang T , Zheng X‐L , Zhao G‐J . Proprotein convertase furin/PCSK3 and atherosclerosis: new insights and potential therapeutic targets. Atherosclerosis. 2017;262:163‐170. 28400053 10.1016/j.atherosclerosis.2017.04.005

[33] Abe SK , Inoue M . Green tea and cancer and cardiometabolic diseases: a review of the current epidemiological evidence. Eur J Clin Nutr. 2021;75(6):865‐876. 32820240 10.1038/s41430-020-00710-7 PMC8189915

[34] MacKintosh ML , Crosbie EJ . Prevention strategies in endometrial carcinoma. Curr Oncol Rep. 2018;20:1‐8. 30426278 10.1007/s11912-018-0747-1 PMC6244901

[35] Tzenios N , Chahine M , Tazanios M . Obesity and endometrial cancer: the role insulin resistance and adipokines. Spec J Med Acad Other Life Sci. 2023;1(2). doi:10.58676/sjmas.v1i2.12

[36] Sacerdote C , Ricceri F . Epidemiological dimensions of the association between type 2 diabetes and cancer: a review of observational studies. Diabetes Res Clin Pract. 2018;143:369‐377. 29596949 10.1016/j.diabres.2018.03.002

[37] Chu D , Wu J , Wang K , et al. Effect of metformin use on the risk and prognosis of endometrial cancer: a systematic review and meta‐analysis. BMC Cancer. 2018;18(1):1‐11. 29669520 10.1186/s12885-018-4334-5 PMC5907461

[38] Njoku K , Abiola J , Russell J , Crosbie EJ . Endometrial cancer prevention in high‐risk women. Best Pract Res Clin Obstet Gynaecol. 2020;65:66‐78. 32107136 10.1016/j.bpobgyn.2019.12.005

[39] Ding S , Madu CO , Lu Y . The impact of hormonal imbalances associated with obesity on the incidence of endometrial cancer in postmenopausal women. J Cancer. 2020;11(18):5456‐5465. 32742493 10.7150/jca.47580 PMC7391192

[40] He Z , Khatib A‐M , Creemers JW . The proprotein convertase furin in cancer: more than an oncogene. Oncogene. 2022;41(9):1252‐1262. 34997216 10.1038/s41388-021-02175-9

[41] McMahon S , Grondin F , McDonald PP , Richard DE , Dubois CM . Hypoxia‐enhanced expression of the proprotein convertase furin is mediated by hypoxia‐inducible factor‐1: impact on the bioactivation of proproteins. J Biol Chem. 2005;280(8):6561‐6569. 15611046 10.1074/jbc.M413248200

[42] Niland S , Riscanevo AX , Eble JA . Matrix metalloproteinases shape the tumor microenvironment in cancer progression. Int J Mol Sci. 2021;23(1):146. 35008569 10.3390/ijms23010146 PMC8745566

[43] Castro F , Cardoso AP , Gonçalves RM , Serre K , Oliveira MJ . Interferon‐gamma at the crossroads of tumor immune surveillance or evasion. Front Immunol. 2018;9:847. 29780381 10.3389/fimmu.2018.00847 PMC5945880

[44] Hipp MM , Shepherd D , Gileadi U , et al. Processing of human toll‐like receptor 7 by furin‐like proprotein convertases is required for its accumulation and activity in endosomes. Immunity. 2013;39(4):711‐721. 24138882 10.1016/j.immuni.2013.09.004 PMC4839496

[45] He Z , Khatib A‐M , Creemers JW . Loss of the proprotein convertase Furin in T cells represses mammary tumorigenesis in oncogene‐driven triple negative breast cancer. Cancer Lett. 2020;484:40‐49. 32389711 10.1016/j.canlet.2020.05.001

[46] He Z , Thorrez L , Siegfried G , et al. The proprotein convertase furin is a pro‐oncogenic driver in KRAS and BRAF driven colorectal cancer. Oncogene. 2020;39(17):3571‐3587. 32139876 10.1038/s41388-020-1238-z

[47] Page RE , Klein‐Szanto AJ , Litwin S , et al. Increased expression of the pro‐protein convertase furin predicts decreased survival in ovarian cancer. Anal Cell Pathol. 2007;29(4):289‐299. 10.1155/2007/930321 PMC4617813 17641413

[48] Chen C , Gupta P , Parashar D , et al. ERBB3‐induced furin promotes the progression and metastasis of ovarian cancer via the IGF1R/STAT3 signaling axis. Oncogene. 2020;39(14):2921‐2933. 32029900 10.1038/s41388-020-1194-7 PMC7346970

[49] Ahmed BS , Aljebori HA , Al‐sudani IM . MicroRNAs 301a and 93 biomarkers for endometrial cancer. Indian J Public Health Res Dev. 2019;10(2):740.

[50] Bassi DE , Mahloogi H , De Cicco RL , Klein‐Szanto A . Increased furin activity enhances the malignant phenotype of human head and neck cancer cells. Am J Pathol. 2003;162(2):439‐447. 12547702 10.1016/s0002-9440(10)63838-2 PMC1851171

[51] Dawood SA , Jwad MA , Hussaini HA . A comparison between the effect of vaginal sildenafil versus oral estradiol valerate on ultrasound parameters of endometrial receptivity and follicular growth in Iraqi females receiving letrozole for induction of ovulation. Iraqi J Embryos Infertil Res. 2020;10(1):35‐50.

[52] Ke JY , Yang J , Li J , Xu Z , Li MQ , Zhu ZL . Baicalein inhibits FURIN‐MT1‐MMP‐mediated invasion of ectopic endometrial stromal cells in endometriosis possibly by reducing the secretion of TGFB1. Am J Reprod Immunol. 2021;85(3):e13344. 32910833 10.1111/aji.13344

[53] Zakrzewski PK . Canonical TGFβ signaling and its contribution to endometrial cancer development and progression—underestimated target of anticancer strategies. J Clin Med. 2021;10(17):3900. 34501347 10.3390/jcm10173900 PMC8432036

[54] Dubois CM , Blanchette F , Laprise M‐H , Leduc R , Grondin F , Seidah NG . Evidence that furin is an authentic transforming growth factor‐β1‐converting enzyme. Am J Pathol. 2001;158(1):305‐316. 11141505 10.1016/s0002-9440(10)63970-3 PMC1850265

[55] Bernot D , Stalin J , Stocker P , et al. Plasminogen activator inhibitor 1 is an intracellular inhibitor of furin proprotein convertase. J Cell Sci. 2011;124(8):1224‐1230. 21406565 10.1242/jcs.079889

[56] Batiha GE‐S , Al‐kuraishy HM , Al‐Maiahy TJ , et al. Plasminogen activator inhibitor 1 and gestational diabetes: the causal relationship. Diabetol Metab Syndr. 2022;14(1):127. doi:10.1186/s13098-022-00900-2 36076264 PMC9454110

[57] Lin L‐L , Kost ER , Lin C‐L , et al. PAI‐1‐dependent inactivation of SMAD4‐modulated junction and adhesion complex in obese endometrial cancer. Cell Rep. 2020;33(2):108253. 33053339 10.1016/j.celrep.2020.108253 PMC7641039

[58] Al‐kuraishy HM , Al‐Gareeb AI , Al‐Harcan NAH , Alexiou A , Batiha G . Tranexamic acid and plasminogen/plasmin glaring paradox in COVID‐19. Endocr Metab Immune Disord Drug Targets. 2023;23(1):35‐45. 35927893 10.2174/1871530322666220801102402

[59] Hagiwara S , Murakumo Y , Mii S , et al. Processing of CD109 by furin and its role in the regulation of TGF‐β signaling. Oncogene. 2010;29(15):2181‐2191. 20101215 10.1038/onc.2009.506

[60] Zhang JM , Hashimoto M , Kawai K , et al. CD109 expression in squamous cell carcinoma of the uterine cervix. Pathol Int. 2005;55(4):165‐169. doi:10.1111/j.1440-1827.2005.01807.x 15826242

[61] Qi R , Dong F , Liu Q , Murakumo Y , Liu J . CD109 and squamous cell carcinoma. J Transl Med. 2018;16(1):88. doi:10.1186/s12967-018-1461-3 29625613 PMC5889571

[62] Koh HM , Lee HJ , Kim DC . Usefulness of CD109 expression as a prognostic biomarker in patients with cancer: a systematic review and meta‐analysis. Medicine. 2021;100(11):e25006. doi:10.1097/md.0000000000025006 33725975 PMC7982172

[63] Martin JH , Mohammed R , Delforce SJ , et al. Role of the prorenin receptor in endometrial cancer cell growth. Oncotarget. 2022;13:587‐599. doi:10.18632/oncotarget.28224 35401936 PMC8986267

[64] Delforce SJ , Lumbers ER , Corbisier de Meaultsart C , et al. Expression of renin‐angiotensin system (RAS) components in endometrial cancer. Endocr Connect. 2017;6(1):9‐19. doi:10.1530/ec-16-0082 27956412 PMC5302162

[65] Schefe JH , Menk M , Reinemund J , et al. A novel signal transduction cascade involving direct physical interaction of the renin/prorenin receptor with the transcription factor promyelocytic zinc finger protein. Circ Res. 2006;99(12):1355‐1366. doi:10.1161/01.RES.0000251700.00994.0d 17082479

[66] Al‐kuraishy HM , Hussien NR , Al‐Naimi MS , Al‐Buhadily AK , Al‐Gareeb AI , Lungnier C . Renin–Angiotensin system and fibrinolytic pathway in COVID‐19: one‐way skepticism. Biomed Biotechnol Res J. 2020;4(5):33.

[67] Al‐kuraishy HM , Al‐Gareeb AI , Mostafa‐Hedeab G , et al. Effects of β‐blockers on the sympathetic and cytokines storms in Covid‐19. Front Immunol. 2021;12:749291. 34867978 10.3389/fimmu.2021.749291 PMC8637815

[68] Al‐kuraishy HM , Al‐Gareeb AI , Abdullah SM , Cruz‐Martins N , Batiha GE‐S . Case report: hyperbilirubinemia in gilbert syndrome attenuates Covid‐19‐induced metabolic disturbances. Front Cardiovasc Med. 2021;8:642181. 33681310 10.3389/fcvm.2021.642181 PMC7925614

[69] Alkuraishy HM , Al‐Gareeb AI , Waheed HJ . Lipoprotein‐associated phospholipase A2 is linked with poor cardio‐metabolic profile in patients with ischemic stroke: a study of effects of statins. J Neurosci Rural Pract. 2018;9(4):496‐503. 30271040 10.4103/jnrp.jnrp_97_18 PMC6126307

[70] Pringle KG , Delforce SJ , Wang Y , et al. Renin–angiotensin system gene polymorphisms and endometrial cancer. Endocr Connect. 2016;5(3):128‐135. doi:10.1530/ec-15-0112 27068935 PMC5002951

[71] Ronquist G , Rodríguez LA , Ruigómez A , et al. Association between captopril, other antihypertensive drugs and risk of prostate cancer. Prostate. 2004;58(1):50‐56. doi:10.1002/pros.10294 14673952

[72] Alkazmi L , Al‐kuraishy HM , Batiha GE‐S , et al. Roxadustat for SARS‐CoV‐2 infection: old signaling raised new hopes. Drugs R&D. 2022;22:183‐186. doi:10.1007/s40268-022-00397-0 PMC9403957 36006604

[73] Cousin C , Bracquart D , Contrepas A , Corvol P , Muller L , Nguyen G . Soluble form of the (pro)renin receptor generated by intracellular cleavage by furin is secreted in plasma. Hypertension. 2009;53(6):1077‐1082. doi:10.1161/hypertensionaha.108.127258 19380613

[74] Yoshikawa A , Aizaki Y , Kusano K , et al. The (pro)renin receptor is cleaved by ADAM19 in the Golgi leading to its secretion into extracellular space. Hypertens Res. 2011;34(5):599‐605. doi:10.1038/hr.2010.284 21270819

[75] Schwarz J , Broder C , Helmstetter A , et al. Short‐term TNFα shedding is independent of cytoplasmic phosphorylation or furin cleavage of ADAM17. Biochim Biophys Acta. 2013;1833(12):3355‐3367. doi:10.1016/j.bbamcr.2013.10.005 24135057

[76] Al‐kuraishy HM , Al‐Gareeb AI , Waheed HJ , Al‐Maiahy TJ . Differential effect of metformin and/or glyburide on apelin serum levels in patients with type 2 diabetes mellitus: concepts and clinical practice. J Adv Pharm Technol Res. 2018;9(3):80‐86. 30338233 10.4103/japtr.JAPTR_273_18 PMC6174705

[77] Wang Y , Dai C , Zhou C , et al. Benzotriazole enhances cell invasive potency in endometrial carcinoma through CTBP1‐mediated epithelial‐mesenchymal transition. Cell Physiol Biochem. 2017;44(6):2357‐2367. doi:10.1159/000486123 29262396

[78] Xu Q , Ying M , Chen G , et al. ADAM17 is associated with EMMPRIN and predicts poor prognosis in patients with uterine cervical carcinoma. Tumour Biol. 2014;35(8):7575‐7586. doi:10.1007/s13277-014-1990-1 24793016

[79] Swärd P , Rosengren BE , Jehpsson L , Karlsson MK . Association between circulating furin levels, obesity and pro‐inflammatory markers in children. Acta Paediatr. 2021;110(6):1863‐1868. doi:10.1111/apa.15774 33486829

[80] Babalghith AO , Al‐kuraishy HM , Al‐Gareeb AI , et al. The potential role of growth differentiation factor 15 in COVID‐19: a corollary subjective effect or not? Diagnostics. 2022;12(9):2051. 36140453 10.3390/diagnostics12092051 PMC9497461

[81] Yakala GK , Cabrera‐Fuentes HA , Crespo‐Avilan GE , et al. FURIN inhibition reduces vascular remodeling and atherosclerotic lesion progression in mice. Arterioscler Thromb Vasc Biol. 2019;39(3):387‐401. doi:10.1161/atvbaha.118.311903 30651003 PMC6393193

[82] Batiha GE‐S , Olatunde A , El‐Mleeh A , et al. Bioactive compounds, pharmacological actions, and pharmacokinetics of wormwood (*Artemisia absinthium*). Antibiotics. 2020;9(6):353. 32585887 10.3390/antibiotics9060353 PMC7345338

[83] Cordova ZM , Grönholm A , Kytölä V , et al. Myeloid cell expressed proprotein convertase FURIN attenuates inflammation. Oncotarget. 2016;7(34):54392‐54404. doi:10.18632/oncotarget.11106 27527873 PMC5342350

[84] Batiha GE‐S , Tayebwa DS , Beshbishy AM , N'Da DD , Yokoyama N , Igarashi I . Inhibitory effects of novel ciprofloxacin derivatives on the growth of four *Babesia* species and *Theileria equi* . Parasitol Res. 2020;119(9):3061‐3073. 32677000 10.1007/s00436-020-06796-z

[85] Pesu M , Watford WT , Wei L , et al. T‐cell‐expressed proprotein convertase furin is essential for maintenance of peripheral immune tolerance. Nature. 2008;455(7210):246‐250. doi:10.1038/nature07210 18701887 PMC2758057

[86] Oksanen A , Aittomäki S , Jankovic D , et al. Proprotein convertase FURIN constrains Th2 differentiation and is critical for host resistance against *Toxoplasma gondii* . J Immunol. 2014;193(11):5470‐5479. doi:10.4049/jimmunol.1401629 25355923 PMC4261955

[87] Lin H , Ah Kioon MD , Lalou C , et al. Protective role of systemic furin in immune response‐induced arthritis. Arthritis Rheum. 2012;64(9):2878‐2886. doi:10.1002/art.34523 22605541

[88] Modugno F , Ness RB , Chen C , Weiss NS . Inflammation and endometrial cancer: a hypothesis. Cancer Epidemiol Biomarkers Prev. 2005;14(12):2840‐2847. doi:10.1158/1055-9965.epi-05-0493 16364998

[89] Morita I . Distinct functions of COX‐1 and COX‐2. Prostaglandins Other Lipid Mediat. 2002;68‐69:165‐175. doi:10.1016/s0090-6980(02)00029-1 12432916

[90] Al‐kuraishy HM , Al‐Gareeb AI , Saad HM , Batiha GE‐S . Hippo‐YAP signaling and SARS‐CoV‐2 infection: a new mechanistic pathway. Cell Stress Chaperones. 2023;28(2):121‐123. doi:10.1007/s12192-023-01327-y 36752973 PMC9907175

[91] Batiha GE‐S , Alqahtani A , Ojo OA , et al. Biological properties, bioactive constituents, and pharmacokinetics of some *Capsicum* spp. and capsaicinoids. Int J Mol Sci. 2020;21(15):5179. 32707790 10.3390/ijms21155179 PMC7432674

[92] Lin Y , Bai L , Chen W , Xu S . The NF‐κB activation pathways, emerging molecular targets for cancer prevention and therapy. Expert Opin Ther Targets. 2010;14(1):45‐55. doi:10.1517/14728220903431069 20001209 PMC3043547

[93] Alomair BM , Al‐kuraishy HM , Al‐Gareeb AI , et al. Montelukast and acute coronary syndrome: the endowed drug. Pharmaceuticals. 2022;15(9):1147. 36145367 10.3390/ph15091147 PMC9500901

[94] Wang X , Wei Z , Tang Z , et al. IL‐37bΔ1‐45 suppresses the migration and invasion of endometrial cancer cells by targeting the Rac1/NF‐κB/MMP2 signal pathway. Lab Invest. 2021;101(6):760‐774. doi:10.1038/s41374-021-00544-2 33753880

[95] Brant KA , Leikauf GD . Dysregulation of FURIN by prostaglandin‐endoperoxide synthase 2 in lung epithelial NCI‐H292 cells. Mol Carcinog. 2014;53(3):192‐200. doi:10.1002/mc.21963 23065687

[96] Yang D , Yao M , Yan Y , et al. Deoxycholic acid upregulates serum golgi protein 73 through activating NF‐κB pathway and destroying Golgi structure in liver disease. Biomolecules. 2021;11(2):205. doi:10.3390/biom11020205 33540642 PMC7913056

[97] Takiuchi T , Blake EA , Matsuo K , Sood AK , Brasky TM . Aspirin use and endometrial cancer risk and survival. Gynecol Oncol. 2018;148(1):222‐232. doi:10.1016/j.ygyno.2017.10.026 29132875 PMC7531033

[98] El‐Saber Batiha G , Al‐Gareeb AI , Saad HM , Al‐kuraishy HM . COVID‐19 and corticosteroids: a narrative review. Inflammopharmacology. 2022;30:1189‐1205. doi:10.1007/s10787-022-00987-z 35562628 PMC9106274

[99] Wang Y , Zhao J , Chen X , Zhang F , Li X . Aspirin use and endometrial cancer risk: a meta‐analysis and systematic review. Ann Transl Med. 2020;8(7):461. doi:10.21037/atm.2020.03.125 32395505 PMC7210134

[100] Coppola JM , Bhojani MS , Ross BD , Rehemtulla A . A small‐molecule furin inhibitor inhibits cancer cell motility and invasiveness. Neoplasia. 2008;10(4):363‐370. 18392131 10.1593/neo.08166 PMC2288536

[101] Cameron A , Appel J , Houghten RA , Lindberg I . Polyarginines are potent furin inhibitors. J Biol Chem. 2000;275(47):36741‐36749. 10958789 10.1074/jbc.M003848200

[102] Marinello PC , Panis C , Silva TN , et al. Oxidative stress and TGF‐β1 induction by metformin in MCF‐7 and MDA‐MB‐231 human breast cancer cells are accompanied with the downregulation of genes related to cell proliferation, invasion and metastasis. Pathol Res Pract. 2020;216(10):153135. 32853957 10.1016/j.prp.2020.153135

[103] Tan BK , Adya R , Chen J , Lehnert H , Cassia LJS , Randeva HS . Metformin treatment exerts antiinvasive and antimetastatic effects in human endometrial carcinoma cells. J Clin Endocrinol Metabol. 2011;96(3):808‐816. 10.1210/jc.2010-1803 21190977

[104] Zhuo Z , Wang A , Yu H . Metformin targeting autophagy overcomes progesterone resistance in endometrial carcinoma. Arch Gynecol Obstet. 2016;294:1055‐1061. 27402506 10.1007/s00404-016-4148-0

[105] Mitsuhashi A , Kawasaki Y , Hori M , Fujiwara T , Hanaoka H , Shozu M . Medroxyprogesterone acetate plus metformin for fertility‐sparing treatment of atypical endometrial hyperplasia and endometrial carcinoma: trial protocol for a prospective, randomised, open, blinded‐endpoint design, dose‐response trial (FELICIA trial). BMJ Open. 2020;10(2):e035416. 10.1136/bmjopen-2019-035416 PMC7050341 32114477

[106] Clement NS , Oliver TR , Shiwani H , Saner JR , Mulvaney CA , Atiomo W . Metformin for endometrial hyperplasia: a Cochrane protocol. BMJ Open. 2016;6(8):e013385. 10.1136/bmjopen-2016-013385 PMC5013431 27531741

[107] Zhao Y , Sun H , Feng M , et al. Metformin is associated with reduced cell proliferation in human endometrial cancer by inbibiting PI3K/AKT/mTOR signaling. Gynecol Endocrinol. 2018;34(5):428‐432. 29182407 10.1080/09513590.2017.1409714

[108] Guo H , Kong W , Zhang L , et al. Reversal of obesity‐driven aggressiveness of endometrial cancer by metformin. Am J Cancer Res. 2019;9(10):2170‐2193. 31720081 PMC6834476

[109] Chae‐Kim J , Garg G , Gavrilova‐Jordan L , Blake LE , Wu Q , Hayslip CC . Outcomes of women treated with progestin and metformin for atypical endometrial hyperplasia and early endometrial cancer: a systematic review and meta‐analysis. Int J Gynecol Cancer. 2021;31(12):1499‐1505. 34785524 10.1136/ijgc-2021-002699

[110] Tabrizi AD , Melli MS , Foroughi M , Ghojazadeh M , Bidadi S . Antiproliferative effect of metformin on the endometrium—a clinical trial. Asian Pac J Cancer Prev. 2014;15(23):10067‐10070. 25556427 10.7314/apjcp.2014.15.23.10067

[111] Al‐kuraishy HM , Al‐Gareeb AI , Alexiou A , et al. Pros and cons for statins use and risk of Parkinson's disease: an updated perspective. Pharmacol Res Perspect. 2023;11(2):e01063. 36811160 10.1002/prp2.1063 PMC9944858

[112] Al‐kuraishy HM , Al‐Gareeb AI , Hussien NR , Al‐Naimi MS , Rasheed HA . Statins an oft‐prescribed drug is implicated in peripheral neuropathy: the time to know more. J Pak Med Assoc. 2019;69(8):S108‐S112. 31603889

[113] Al‐kuraishy HM , Al‐Gareeb AI . Acylation‐stimulating protein is a surrogate biomarker for acute myocardial infarction: role of statins. J Lab Physicians. 2017;9(3):163‐169. 28706385 10.4103/0974-2727.208263 PMC5496293

[114] Sawaguchi J , Saeki Y , Oda M , et al. The circulating furin‐cleaved/mature PCSK9 ratio has a potential prognostic significance in statin‐naïve patients with acute ST elevation myocardial infarction. Atheroscler Plus. 2022;50:50‐56. 36643795 10.1016/j.athplu.2022.09.002 PMC9833232

[115] Kuyama N , Kataoka Y , Takegami M , et al. Circulating mature PCSK9 level predicts diminished response to statin therapy. J Am Heart Assoc. 2021;10(11):e019525. 33998287 10.1161/JAHA.120.019525 PMC8483520

[116] Yu C , Wang G , Liu Q , et al. Host antiviral factors hijack furin to block SARS‐CoV‐2, ebola virus, and HIV‐1 glycoproteins cleavage. Emerging Microbes Infect. 2023;12(1):2164742. 10.1080/22221751.2022.2164742 PMC9897805 36591809

[117] Wang H , Yuan Z , Pavel MA , et al. The role of high cholesterol in age‐related COVID19 lethality. *bioRxiv* . 2020.

[118] Hafizz AMHA , Zin RRM , Aziz NHA , Kampan NC , Shafiee MN . Beyond lipid‐lowering: role of statins in endometrial cancer. Mol Biol Rep. 2020;47:8199‐8207. 32897522 10.1007/s11033-020-05760-5

[119] Feng J‐L , Dixon‐Suen SC , Jordan SJ , Webb PM . Is there sufficient evidence to recommend women diagnosed with endometrial cancer take a statin: results from an Australian record‐linkage study. Gynecol Oncol. 2021;161(3):858‐863. 33846016 10.1016/j.ygyno.2021.04.001

[120] Urpilainen E , Ahtikoski A , Arima R , Puistola U , Karihtala P . No association between statin use and the prognosis of endometrial cancer in women with type 2 diabetes. Front Pharmacol. 2021;12:621180. 34054515 10.3389/fphar.2021.621180 PMC8155720

[121] Nevadunsky NS , Van Arsdale A , Strickler HD , et al. Association between statin use and endometrial cancer survival. Obstet Gynecol. 2015;126(1):144‐150. 26241267 10.1097/AOG.0000000000000926

[122] Desai P , Wallace R , Anderson ML , et al. An analysis of the association between statin use and risk of endometrial and ovarian cancers in the Women's Health Initiative. Gynecol Oncol. 2018;148(3):540‐546. 29422345 10.1016/j.ygyno.2018.01.006 PMC5896309

[123] Akinwunmi B , Vitonis AF , Titus L , Terry KL , Cramer DW . Statin therapy and association with ovarian cancer risk in the New England case control (NEC) study. Int J Cancer. 2019;144(5):991‐1000. 30006925 10.1002/ijc.31758 PMC6320710

[124] Wang Y , Ren F , Song Z , Chen P , Liu S , Ouyang L . Statin use and the risk of ovarian and endometrial cancers: a meta‐analysis. BMC Cancer. 2019;19:1‐10. 31340777 10.1186/s12885-019-5954-0 PMC6657066

[125] Tuli HS , Aggarwal V , Kaur J , et al. Baicalein: a metabolite with promising antineoplastic activity. Life Sci. 2020;259:118183. 32781058 10.1016/j.lfs.2020.118183

[126] Selvaraj LK , Jeyabalan S , Wong LS , et al. Baicalein prevents stress‐induced anxiety behaviors in zebrafish model. Front Pharmacol. 2022;13:4516. 10.3389/fphar.2022.990799 PMC9659741 36386131

[127] Verma E , Kumar A , Daimary UD , et al. Potential of baicalein in the prevention and treatment of cancer: a scientometric analyses based review. J Funct Foods. 2021;86:104660.

[128] Mcglorthan L , Syed V . Baicalein attenuates endometrial cancer growth by suppressing the ARF6. Cancer Res. 2022;82(12_Supplement):1864.

[129] Li K , Diakite D , Austin J , et al. The flavonoid baicalein negatively regulates progesterone target genes in the uterus in vivo. J Nat Prod. 2021;85(1):237‐247. 34935393 10.1021/acs.jnatprod.1c01008 PMC9164990

[130] Kavandi L , Lee LR , Bokhari AA , et al. The Chinese herbs *Scutellaria baicalensis* and *Fritillaria cirrhosa* target NFκB to inhibit proliferation of ovarian and endometrial cancer cells. Mol Carcinog. 2015;54(5):368‐378. 24249479 10.1002/mc.22107

[131] Dinda B , Dinda M , Dinda S , De UC . An overview of anti‐SARS‐CoV‐2 and anti‐inflammatory potential of baicalein and its metabolite baicalin: insights into molecular mechanisms. Eur J Med Chem. 2023;258:115629. 37437351 10.1016/j.ejmech.2023.115629

